# The role of hypoxia in intestinal inflammation

**DOI:** 10.1186/s40348-016-0030-1

**Published:** 2016-01-26

**Authors:** Yatrik M. Shah

**Affiliations:** 1 Department of Molecular & Integrative Physiology, University of Michigan Medical School, Ann Arbor, MI 48109 USA; 2 Department of Internal medicine, Division of Gastroenterology, University of Michigan Medical School, Ann Arbor, MI 48109 USA

**Keywords:** Hypoxia, IBD, Ulcerative colitis, Crohn’s disease, Colon cancer, HIF-1α, HIF-2α

## Abstract

Inflammatory bowel disease (IBD) is a chronic relapsing inflammatory disease of the intestine. IBD is a multifactorial disorder, and IBD-associated genes are critical in innate immune response, inflammatory response, autophagy, and epithelial barrier integrity. Moreover, epithelial oxygen tension plays a critical role in intestinal inflammation and resolution in IBD. The intestines have a dynamic and rapid fluctuation in cellular oxygen tension, which is dysregulated in IBD. Intestinal epithelial cells have a steep oxygen gradient where the tips of the villi are hypoxic and the oxygenation increases at the base of the villi. IBD results in heightened hypoxia throughout the mucosa. Hypoxia signals through a well-conserved family of transcription factors, where hypoxia-inducible factor (HIF)-1α and HIF-2α are essential in maintaining intestinal homeostasis. In inflamed mucosa, HIF-1α increases barrier protective genes, elicits protective innate immune responses, and activates an antimicrobial response through the increase in β-defensins. HIF-2α is essential in maintaining an epithelial-elicited inflammatory response and the regenerative and proliferative capacity of the intestine following an acute injury. HIF-1α activation in colitis leads to a protective response, whereas chronic activation of HIF-2α increases the pro-inflammatory response, intestinal injury, and cancer. In this mini-review, we detail the role of HIF-1α and HIF-2α in intestinal inflammation and injury and therapeutic implications of targeting HIF signaling in IBD.

## Introduction

The intestine is a highly regenerative tissue, which completely renews every 5 to 6 days. The intestinal epithelial cells are critical for digestion, secretion of hormones and mucin, and absorption of nutrients. The epithelial cells are under control by an exquisite signaling cascade (Notch, BMP, Wnt/β-catenin) that maintains proliferation and differentiation of epithelial progenitors and the self-renewal capacity of intestinal epithelial stem cells. In addition, cellular oxygen dynamics are critical in maintaining intestinal homeostasis. The intricate oxygen gradient in the intestine is set up by the rapid propagation of the enteric microbiota just after birth. The microbiota composition of newborns suggests that aerobic and facultative anaerobic bacteria consume luminal oxygen, which allows the growth of obligate anaerobes and establishes an anoxic lumen [1]. The anoxic lumen in part establishes the oxygen gradient of the intestinal epithelium, as cells directly adjacent to the lumen are hypoxic relative to the cells that are close to the base of the crypts [2]. Moreover, microbiota-derived short-chain fatty acids regulate oxygen consumption in intestinal epithelial cells [3]. Dysregulation of oxygen gradients is observed in inflammatory bowel disease (IBD). IBD is divided into two major subgroups: ulcerative colitis (UC) and Crohn’s disease (CD). The precise etiology of IBD is unknown. However, oxygen signaling plays an important function in the inflammatory and injury response.

### Oxygen sensing and signaling in the intestine

**Fig. 1:**
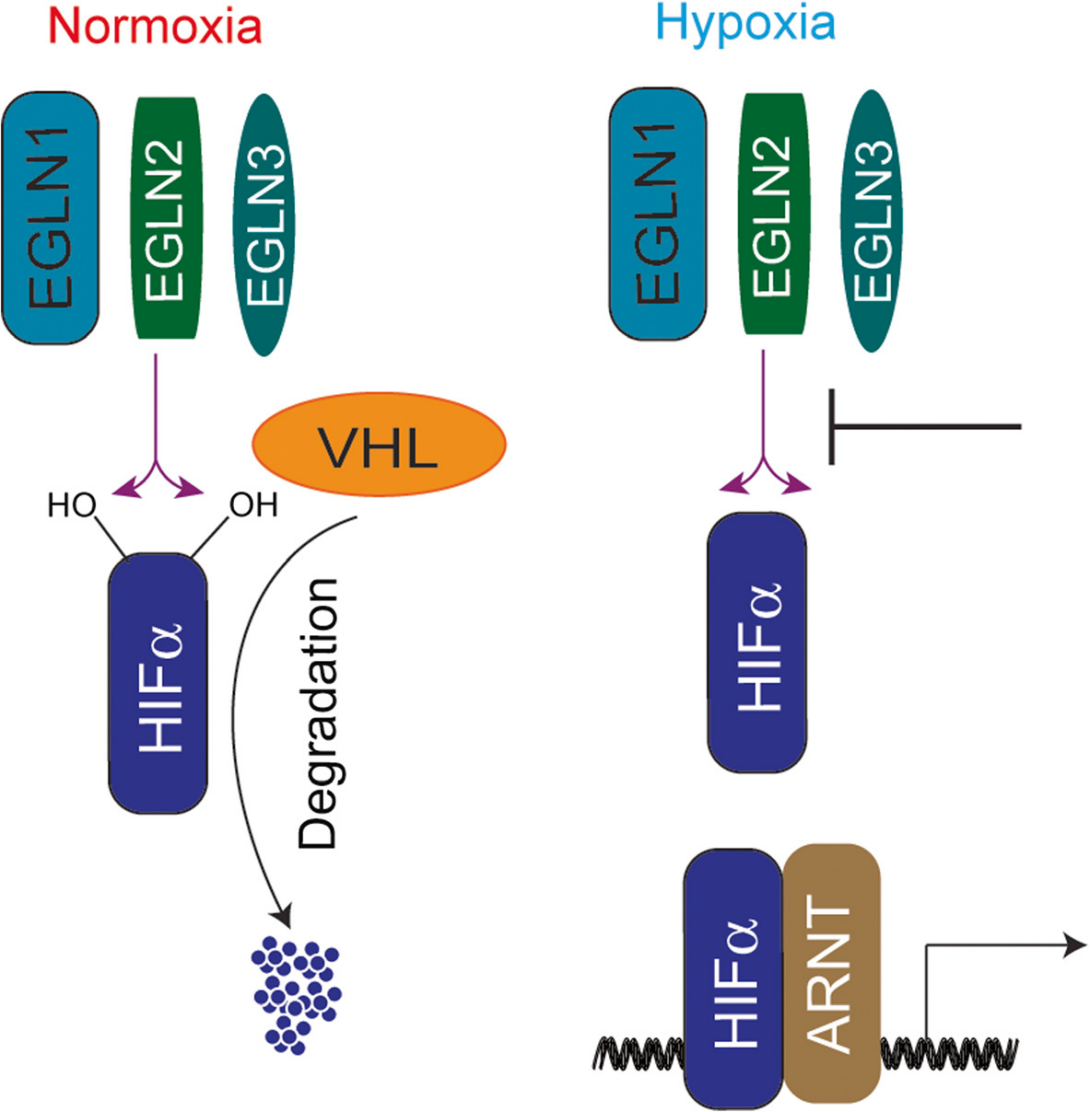
Schematic diagram of oxygen-dependent HIF regulation. In normoxia, HIF-α subunits are hydroxylated on two conserved proline residues by ELGN1, ELGN2, or EGLN3. Hydroxylation of HIF-α subunit leads to rapid degradation through von Hippel-Lindau tumor suppressor protein (VHL) binding and proteasome-mediated degradation. In hypoxia, proline hydroxylation is inhibited resulting in decreased VHL binding and stabilization of protein. Following stabilization, HIF-α subunit forms a heterodimer with (ARNT) leading to activation of HIF target genes. *ARNT* aryl hydrocarbon receptor nuclear translocator, *EGLN* EGL nine homolog, *HIF* hypoxia-inducible factor, *VHL* von Hippel-Lindau tumor suppressor protein

The major transcription factor, which mediates the cellular response to hypoxia, is hypoxia-inducible factor (HIF). HIFs are highly conserved transcription factors that are present in all metazoans. During the divergence of vertebrates, gene duplication led to the emergence of additional α subunits. Mammals contain HIF-1α, HIF-2α, and HIF-3α. HIF-α subunits are regulated through a well-characterized hydroxylation-induced proteasome-mediated degradation during normal cellular oxygen tension. Degradation is mediated by prolyl hydroxylase domain enzymes (PHDs). Mammalian cells contain three PHDs, EGL nine homolog (EGLN)1, EGLN2, and EGLN3. PHDs are α-ketoglutarate dependent dioxygenases that hydroxylate HIF-α subunit on two proline residues. PHD specificity for HIF-α subunits is not clear, but disruption of all three genes is needed for robust HIF-1α and HIF-2α activation in the intestine [4]. Hydroxylation serves as a recognition motif for the von Hippel-Lindau tumor suppressor protein, which recruits an E3 ubiquitin ligase complex leading to rapid proteasomal degradation of HIF-α subunit (Fig. 1). PHDs use molecular oxygen for the hydroxylation reaction, and therefore, in a limited oxygen environment, HIF-α subunit is not hydroxylated leading to stabilization. HIF-α subunit binds to an obligate heterodimer binding partner HIF-1β also called aryl hydrocarbon receptor nuclear translocator (ARNT) and subsequent recruitment to HIF response elements (HREs) present in the promoter of HIF target genes (Fig. 1). It is estimated that in a specific cell type, HIFs bind to roughly 500 sites following hypoxic activation [5].

### Hypoxia and IBD

Using staining techniques to visualize hypoxic foci, a robust increase in hypoxia is observed in mouse models of colitis [2]. Physiological hypoxia as mentioned above is localized to epithelial cells adjacent to the lumen. In colitis, hypoxic staining is observed throughout the mucosa. The precise mechanism for the increase in hypoxia is not clear, but it is likely due to several factors. Inflammation leads to enhanced oxygen consumption of intestinal epithelial cells. Inflammation increases local vasculitis and thus decreasing the oxygen availability to inflamed areas [6]. Recently, it was shown that transmigrating neutrophils can consume local oxygen, thereby enhancing hypoxia in colitis [7]. In addition to hypoxic staining in mouse models, HIF-1α and HIF-2α are highly increased in epithelial cells in UC and CD patients [8]. Currently, the expression and function of HIF-3α have not been thoroughly assessed.

### HIF-1α and HIF-2α targets in IBD

**Fig. 2:**
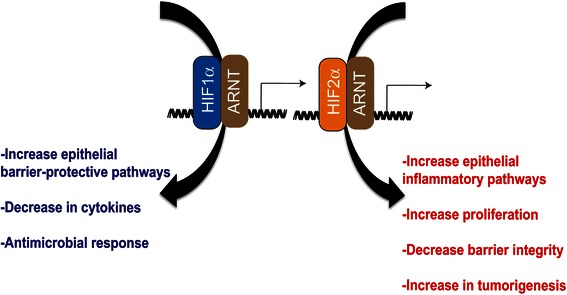
Distinct roles of HIF-1α and HIF-2α in IBD. Activation of HIF-1α increases barrier protective genes and activates a protective innate immune response and antimicrobial response. HIF-2α activation in the intestine leads to an increase in pro-inflammatory mediators and decrease barrier integrity and results in increase susceptibility to colon tumors. *ARNT* aryl hydrocarbon receptor nuclear translocator, *HIF* hypoxia-inducible factor

HIF-1α and HIF-2α can bind to the same canonical HREs. However, through mouse models and cell studies, it is clear that HIF-1α and HIF-2α regulate distinct subset of genes. HIF-1α in intestinal epithelial cells is widely recognized as a major protective factor in IBD. IBD results in a dysregulation of a very complex and intricate network of tight junctions, which are critical in maintaining a barrier that is needed to separate commensal microbiota from the mucosal immune cells. HIF-1α directly regulates several barrier protective genes during injury. Moreover, HIF-1α activation can decrease cytokines and leads to an increase in β-defensins, a critical antimicrobial protein (Fig. 2) [2, 9–13]. PHDs inhibitors, which activate HIF-1α and HIF-2α, are protective in acute colitis models through a HIF-1α-dependent mechanism [12, 14, 15]. However, chronic activation of HIF-2α in intestinal epithelial cells leads to a robust spontaneous intestinal inflammation in a dose-dependent manner [8]. Using mouse models in which HIF-1α and HIF-2α are overexpressed in intestinal epithelial cells demonstrate a distinct function for these transcription factors in IBD. Moderate overexpression of HIF-2α in intestinal epithelial cells does not result in any basal intestinal injury. However, expression of pro-inflammatory mediators is significantly increased, and the mice are highly susceptible to inflammatory injury in mouse models of colitis. Highly overexpressing HIF-2α in intestinal epithelial cells leads to spontaneous colitis, and the mice die at 35 days old from massive intestinal inflammatory disorder [8]. HIF-2α directly regulates a number of pro-inflammatory cytokines including tumor necrosis factor-α, which is essential for HIF-2α-induced inflammation [8]. Moreover, recent work has demonstrated that HIF-2α is essential in barrier function [16, 17]. A chronic increase in HIF-2α leads to high turnover of the tight junction protein occludin, leading to a decrease in barrier integrity [17]. Using similar mouse models, moderate or high overexpression of HIF-1α leads to a decrease in intestinal damage in a colitis model, as expected [18]. Interestingly, activation of HIF-1α does not result in increased tumorigenesis in a colitis-associated colon cancer model, further suggesting that HIF-1α is a good target for colitis [18]. Activation of HIF-2α not only leads to activation of the inflammatory response but there is an increase in tumor number, tumor size, and tumor progression in mouse models of colon cancer [19]. In addition to directly regulating pro-inflammatory mediators and barrier function, HIF-2α is important in the wounding response and proliferation following injury (Fig. 2) [8, 17, 19, 20]. Interestingly, overexpression of both HIF-1α and HIF-2α also leads to heightened inflammatory response suggesting that activation of HIF-1α does not protect the pro-inflammatory response of HIF-2α [8]. This data contradicts the battery of literature showing a protective function of PHD inhibitors in colitis. There maybe several reason for this discrepancy. Chemical inhibition of PHDs leads to a more robust HIF-1α activation rather than HIF-2α activation. This is indeed true for dimethyloxaloylglycine, a commonly used PHD inhibitor, where doses enough for HIF-1α activation do not lead to significant increase in HIF-2α-specific targets [8]. Also, PHD inhibitors may lead to more pulsatile activation of HIF-1α and HIF-2α rather than chronic high increase in HIF-2α, which leads to inflammatory injury. Lastly, the temporal regulation of HIF-1α and HIF-2α has not been critically assessed in chronic models of colitis. HIF-1α is regulated in a cyclical manner through a negative feedback loop mediated by *mir-155*. HIF-1α increases the expression of *miR-155*, which in turn represses HIF-1α expression following sustained hypoxia [21]. Therefore, in IBD, chronic activation of HIF-2α with low HIF-1α expression may promote the pro-inflammatory response and decrease the intestinal barrier integrity leading to heightened inflammation and injury.

### Hypoxia-based therapies

Currently, the pan-PHD inhibitors dimethyloxaloylglycine, FG-4497, and TRC160334 are protective in mouse models of colitis [14, 15, 22]. However, HIF-2α may increase the inflammatory response, and therefore, optimal HIF-based therapies would be pharmacological agents that can specifically increase HIF-1α. AKB-4924 is a PHD inhibitor that results in modest activation of HIF-2α but robustly activates HIF-1α [23]. AKB-4924 increases the antimicrobial response and protective innate immune response. AKB-4924 treatment improves the intestinal barrier integrity and reduces the pro-inflammatory response. The beneficial effects of AKB-4924 were due to intestinal HIF-1α, as disruption of HIF-1α attenuated the protective role [12]. Moreover, HIF-2α inhibitors have been recently identified. HIF-2α (but not HIF-1α) contains ligand-binding cavity, although endogenous substrates have not been identified [24]. This cavity has been targeted for drug development, and several promising highly specific small-molecule inhibitors are identified [25]. Currently, these drugs have not been assessed in mouse models of colitis, but the data suggest that disruption of intestinal epithelial HIF-2α decreases the inflammatory response in colitis [8].

## Conclusion

HIF-1α and HIF-2α play an essential role in IBD. Understanding the temporal regulation of HIF-1α and HIF-2α will be key to design novel and effective HIF-based therapies for IBD. It is likely that both responses are critical in the initiation and resolution of intestinal inflammation. HIF-1α increases the barrier integrity and antimicrobial response, whereas HIF-2α activates pro-inflammatory mediators to elicit an immune response and stimulates epithelial proliferation to promote regeneration. However, more work is needed to understand the dynamic regulation of HIF-1α and HIF-2α in models of chronic colitis.

## References

[1] Albenberg L, Esipova TV, Judge CP, Bittinger K, Chen J, Laughlin A, Grunberg S, Baldassano RN, Lewis JD, Li H, Thom SR, Bushman FD, Vinogradov SA, Wu GD (2014). Correlation between intraluminal oxygen gradient and radial partitioning of intestinal microbiota. Gastroenterology.

[2] Karhausen J, Furuta GT, Tomaszewski JE, Johnson RS, Colgan SP, Haase VH (2004). Epithelial hypoxia-inducible factor-1 is protective in murine experimental colitis. J Clin Invest.

[3] Kelly CJ, Zheng L, Campbell EL, Saeedi B, Scholz CC, Bayless AJ, Wilson KE, Glover LE, Kominsky DJ, Magnuson A, Weir TL, Ehrentraut SF, Pickel C, Kuhn KA, Lanis JM, Nguyen V, Taylor CT, Colgan SP (2015). Crosstalk between microbiota-derived short-chain fatty acids and intestinal epithelial HIF augments tissue barrier function. Cell Host Microbe.

[4] Taniguchi CM, Miao YR, Diep AN, Wu C, Rankin EB, Atwood TF, Xing L, Giaccia AJ (2014) PHD inhibition mitigates and protects against radiation-induced gastrointestinal toxicity via HIF2. Sci Transl Med 6 (236):236ra264. doi:10.1126/scitranslmed.3008523 10.1126/scitranslmed.3008523 PMC4136475 24828078

[5] Schodel J, Mole DR, Ratcliffe PJ (2013). Pan-genomic binding of hypoxia-inducible transcription factors. Biol Chem.

[6] Colgan SP, Taylor CT (2010). Hypoxia: an alarm signal during intestinal inflammation. Nat Rev Gastroenterol Hepatol.

[7] Campbell EL, Bruyninckx WJ, Kelly CJ, Glover LE, McNamee EN, Bowers BE, Bayless AJ, Scully M, Saeedi BJ, Golden-Mason L, Ehrentraut SF, Curtis VF, Burgess A, Garvey JF, Sorensen A, Nemenoff R, Jedlicka P, Taylor CT, Kominsky DJ, Colgan SP (2014). Transmigrating neutrophils shape the mucosal microenvironment through localized oxygen depletion to influence resolution of inflammation. Immunity.

[8] Xue X, Ramakrishnan S, Anderson E, Taylor M, Zimmermann EM, Spence JR, Huang S, Greenson JK, Shah YM (2013). Endothelial PAS domain protein 1 activates the inflammatory response in the intestinal epithelium to promote colitis in mice. Gastroenterology.

[9] Furuta GT, Turner JR, Taylor CT, Hershberg RM, Comerford K, Narravula S, Podolsky DK, Colgan SP (2001). Hypoxia-inducible factor 1-dependent induction of intestinal trefoil factor protects barrier function during hypoxia. J Exp Med.

[10] Louis NA, Hamilton KE, Canny G, Shekels LL, Ho SB, Colgan SP (2006). Selective induction of mucin-3 by hypoxia in intestinal epithelia. J Cell Biochem.

[11] Synnestvedt K, Furuta GT, Comerford KM, Louis N, Karhausen J, Eltzschig HK, Hansen KR, Thompson LF, Colgan SP (2002). Ecto-5′-nucleotidase (CD73) regulation by hypoxia-inducible factor-1 mediates permeability changes in intestinal epithelia. J Clin Invest.

[12] Keely S, Campbell EL, Baird AW, Hansbro PM, Shalwitz RA, Kotsakis A, McNamee EN, Eltzschig HK, Kominsky DJ, Colgan SP (2014). Contribution of epithelial innate immunity to systemic protection afforded by prolyl hydroxylase inhibition in murine colitis. Mucosal Immunol.

[13] Kelly CJ, Glover LE, Campbell EL, Kominsky DJ, Ehrentraut SF, Bowers BE, Bayless AJ, Saeedi BJ, Colgan SP (2013). Fundamental role for HIF-1alpha in constitutive expression of human beta defensin-1. Mucosal Immunol.

[14] Cummins EP, Seeballuck F, Keely SJ, Mangan NE, Callanan JJ, Fallon PG, Taylor CT (2008). The hydroxylase inhibitor dimethyloxalylglycine is protective in a murine model of colitis. Gastroenterology.

[15] Robinson A, Keely S, Karhausen J, Gerich ME, Furuta GT, Colgan SP (2008). Mucosal protection by hypoxia-inducible factor prolyl hydroxylase inhibition. Gastroenterology.

[16] Glover LE, Bowers BE, Saeedi B, Ehrentraut SF, Campbell EL, Bayless AJ, Dobrinskikh E, Kendrick AA, Kelly CJ, Burgess A, Miller L, Kominsky DJ, Jedlicka P, Colgan SP (2013). Control of creatine metabolism by HIF is an endogenous mechanism of barrier regulation in colitis. Proc Natl Acad Sci U S A.

[17] Xie L, Xue X, Taylor M, Ramakrishnan SK, Nagaoka K, Hao C, Gonzalez FJ, Shah YM (2014). Hypoxia-inducible factor/MAZ-dependent induction of caveolin-1 regulates colon permeability through suppression of occludin, leading to hypoxia-induced inflammation. Mol Cell Biol.

[18] Xue X, Ramakrishnan SK, Shah YM (2014). Activation of HIF-1alpha does not increase intestinal tumorigenesis. Am J Physiol Gastrointest Liver Physiol.

[19] Xue X, Taylor M, Anderson E, Hao C, Qu A, Greenson JK, Zimmermann EM, Gonzalez FJ, Shah YM (2012). Hypoxia-inducible factor-2alpha activation promotes colorectal cancer progression by dysregulating iron homeostasis. Cancer Res.

[20] Xue X, Shah YM (2013). Hypoxia-inducible factor-2alpha is essential in activating the COX2/mPGES-1/PGE2 signaling axis in colon cancer. Carcinogenesis.

[21] Bruning U, Cerone L, Neufeld Z, Fitzpatrick SF, Cheong A, Scholz CC, Simpson DA, Leonard MO, Tambuwala MM, Cummins EP, Taylor CT (2011). MicroRNA-155 promotes resolution of hypoxia-inducible factor 1alpha activity during prolonged hypoxia. Mol Cell Biol.

[22] Gupta R, Chaudhary AR, Shah BN, Jadhav AV, Zambad SP, Gupta RC, Deshpande S, Chauthaiwale V, Dutt C (2014). Therapeutic treatment with a novel hypoxia-inducible factor hydroxylase inhibitor (TRC160334) ameliorates murine colitis. Clin Exper Gastroenterol.

[23] Okumura CY, Hollands A, Tran DN, Olson J, Dahesh S, von Kockritz-Blickwede M, Thienphrapa W, Corle C, Jeung SN, Kotsakis A, Shalwitz RA, Johnson RS, Nizet V (2012). A new pharmacological agent (AKB-4924) stabilizes hypoxia inducible factor-1 (HIF-1) and increases skin innate defenses against bacterial infection. J Mol Med.

[24] Wu D, Potluri N, Lu J, Kim Y, Rastinejad F (2015). Structural integration in hypoxia-inducible factors. Nature.

[25] Scheuermann TH, Li Q, Ma HW, Key J, Zhang L, Chen R, Garcia JA, Naidoo J, Longgood J, Frantz DE, Tambar UK, Gardner KH, Bruick RK (2013). Allosteric inhibition of hypoxia inducible factor-2 with small molecules. Nat Chem Biol.

